# Supplementary material for: Inferring a spatial code of cell-cell interactions across a whole animal body
Source: PLoS Comput Biol. 2022 Nov 17;18(11):e1010715. doi: 10.1371/journal.pcbi.1010715 (PMC9714814; doi:10.1371/journal.pcbi.1010715)
Supplement: S1 Text — It also includes N1-4 Fig. (DOCX) [file pcbi.1010715.s001.docx]

**S1 Text**

**Specific ligand-receptor pairs drive an overall cell-cell interaction anticorrelated with spatial distance between cells.**

Since our CCI score is undirected, it can also be compared with spatial properties such as the distance between cells. Under the hypothesis that larger distances should decrease the potential of cells to interact, we expected our CCI scores to be negatively correlated with the Euclidean distances between cells. Thus, we used the distances between cells, calculated by taking the Euclidean distance between cells from a 3D atlas of *C. elegans* (N1A Fig), as our reference data to assess our methodology and assumptions in calculating our CCI score (see ***Computing cell-cell interactions***)

We annotated each cell in the 3D atlas with a corresponding cell type in the scRNA-seq dataset (S6 Table), and computed the minimal Euclidean distances between each pair of cell types (N1B Fig). The minimal distance was used because it would represent the maximal potential that cells have to interact. Thus, we next calculated the Spearman correlation between the CCI score matrix and the Euclidean distance matrix. As expected the correlation coefficient of -0.21 (P-value = 0.0016) was negative. However, the low value may be due to noise introduced by comprehensively incorporating all LR pairs into the computation, which likely includes LR pairs not necessarily encoding spatial information.

We hypothesized there is a subset of key LR pairs most relevant to spatial organization, which could be found by analyzing co-expression of cells interacting at varying proximities. To identify the LR pairs encoding spatial patterning, we ran a genetic algorithm (GA) to maximize the correlation between the CCI score matrix and the Euclidean distance matrix by randomly generating different size subsets of the LR pairs in our complete list (N2A-B Fig). This algorithm was run 100 times, obtaining in each case a different optimal list of LR pairs due to the stochastic nature of this algorithm (N2C Fig). Nevertheless, across all solutions, an average Spearman coefficient of -0.67 ± 0.01 was obtained (shown as an absolute value in N2B Fig) and the maximal correlation resulted in a value of -0.70 (P-value = 1.435 x 10^-35^).

To find a core subset of LR pairs among the 100 subsets obtained by the GA, we clustered LR pairs by their co-occurrence across the GA runs and selected the cluster with members that were simultaneously present in a high fraction of the optimal subsets (N2C-D Fig). This consensus list included 37 LR pairs (S3 Table), here referred to as GA-LR pairs, whose combined appearance seemed to encode proximity across cell-cell interactions, yielding a Spearman coefficient of -0.63 (P-value = 2.629 x 10^-27^) between the CCI score matrix and the Euclidean distance matrix. To test if the correlation stems from the LR pairs in the consensus list, we performed a series of permutation analyses (N3 Fig). We evaluated if the correlation computed with the consensus list was greater than the value from randomly-generated interactions between ligands and receptors in the GA-LR interactions, either by randomly permuting the ligands and the receptors (N3A Fig) or by shuffling their labels to keep the topology of the interactions (N3B Fig). We also subsampled the complete list of LR pairs (S1 Table) to obtain random subsets of similar size to the list of GA-LR pairs (N3C Fig). In each scenario, the randomized lists yielded a smaller negative Spearman correlation than the consensus list (P-value < 0.0001, see N3 Fig).

**Threshold values for the binary-based CCI scores**

We evaluated different threshold values for the binary-based CCI scores including the Bray-Curtis, LR Count and Smillie scores. Specifically, we tested 5, 10, 50, and 100 TPM as the thresholds to assign whether a ligand or receptor is present in a given cell, and then computed the CCI scores with each of the methods aforementioned. To assess the impact of these thresholds, we trained classifiers with the resulting CCI scores in each case as inputs to distinguish whether the interaction occurs in a short-, mid-, or long-range intercellular distance. Here we used the whole list of 245 LR pairs of *C. elegans* to compute the CCI scores. The performance of each scenario of threshold and CCI scoring method was evaluated with a Receiver Operating Characteristic (ROC) curve and its area under the curve (AUC). The thresholds impacted each of the CCI scoring methods in a different manner, even depending on the distance range inferred (N4 Fig). Overall, the best performers were LR Count with 5 TPM, and Bray-Curtis with 10 TPM. Nevertheless, one could use this approach for an optimal parameterization of each of the approaches; however, it is important to consider that the thresholding in these CCI scores seeks to better represent the presence/absence of proteins. Thus, using a threshold that optimizes the performance of the CCI scoring inferring a phenotype could be misleading since the LR pairs considered as active may be false positives. For this reason, we chose 10 TPM as the threshold for all binary-based scores, since this value was previously selected to better represent the protein presence [[1]](https://paperpile.com/c/vrFqdk/e7mra)**.**


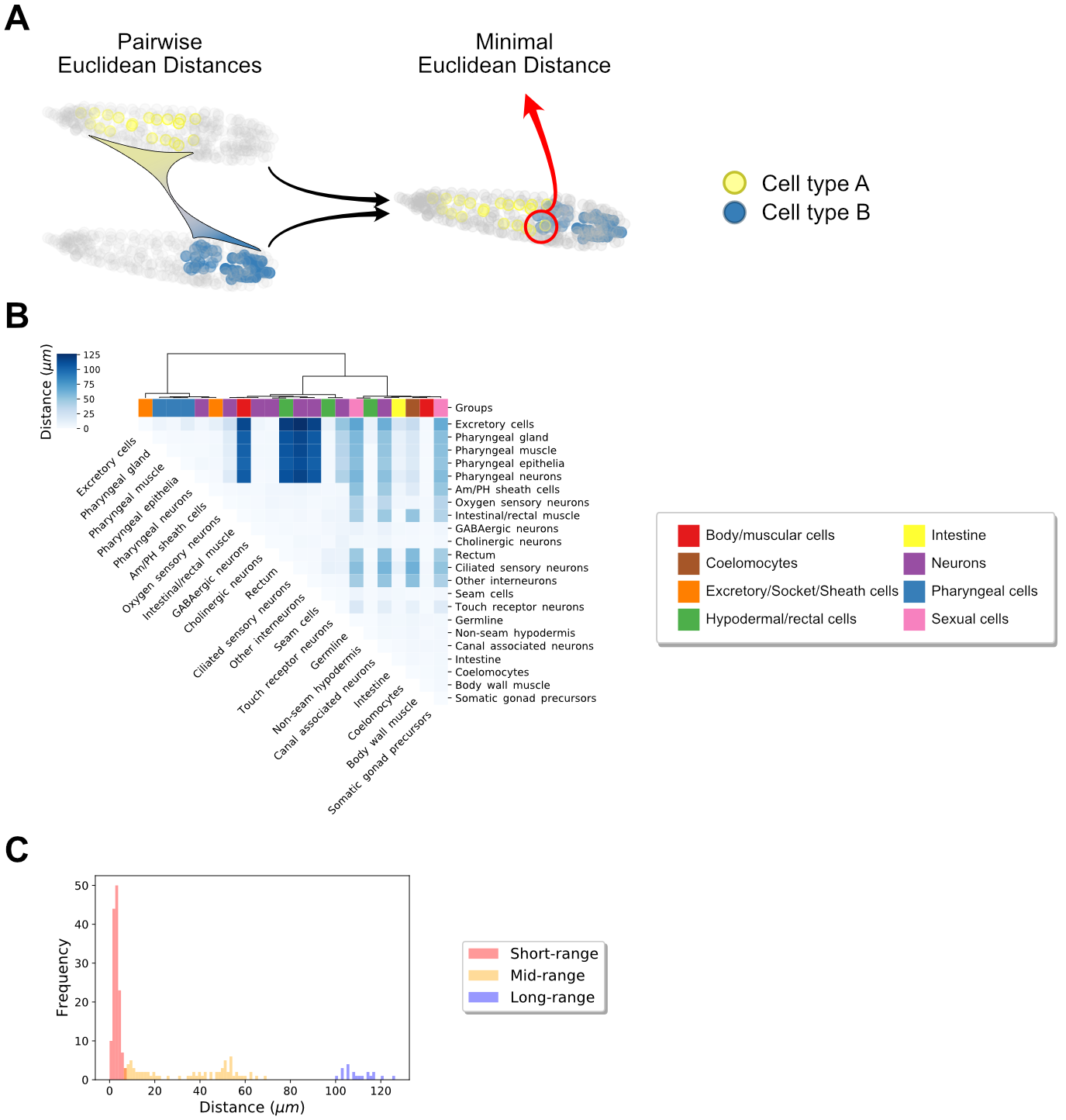


**N1 Fig. Euclidean distances between cells.**

(A) Schematic representation of computing the minimal Euclidean distance for a pair of cell types in *C. elegans*. The distance for all pairs of cells between those belonging to cell type A and those belonging to cell type B is computed; then the minimal one is selected. (B) Heatmap of resulting Euclidean distances among all pairs of cells. Depicted color key defines the eight major cell groups that were previously defined by Cao et al. [[2]](https://paperpile.com/c/vrFqdk/myXwx). (C) Distribution of physical distances between cell types (built with values in the diagonal matrix in (B)). The physical distance between each pair of cell types was classified into short-, mid- and long-range distances by using a three components Gaussian mixed model.


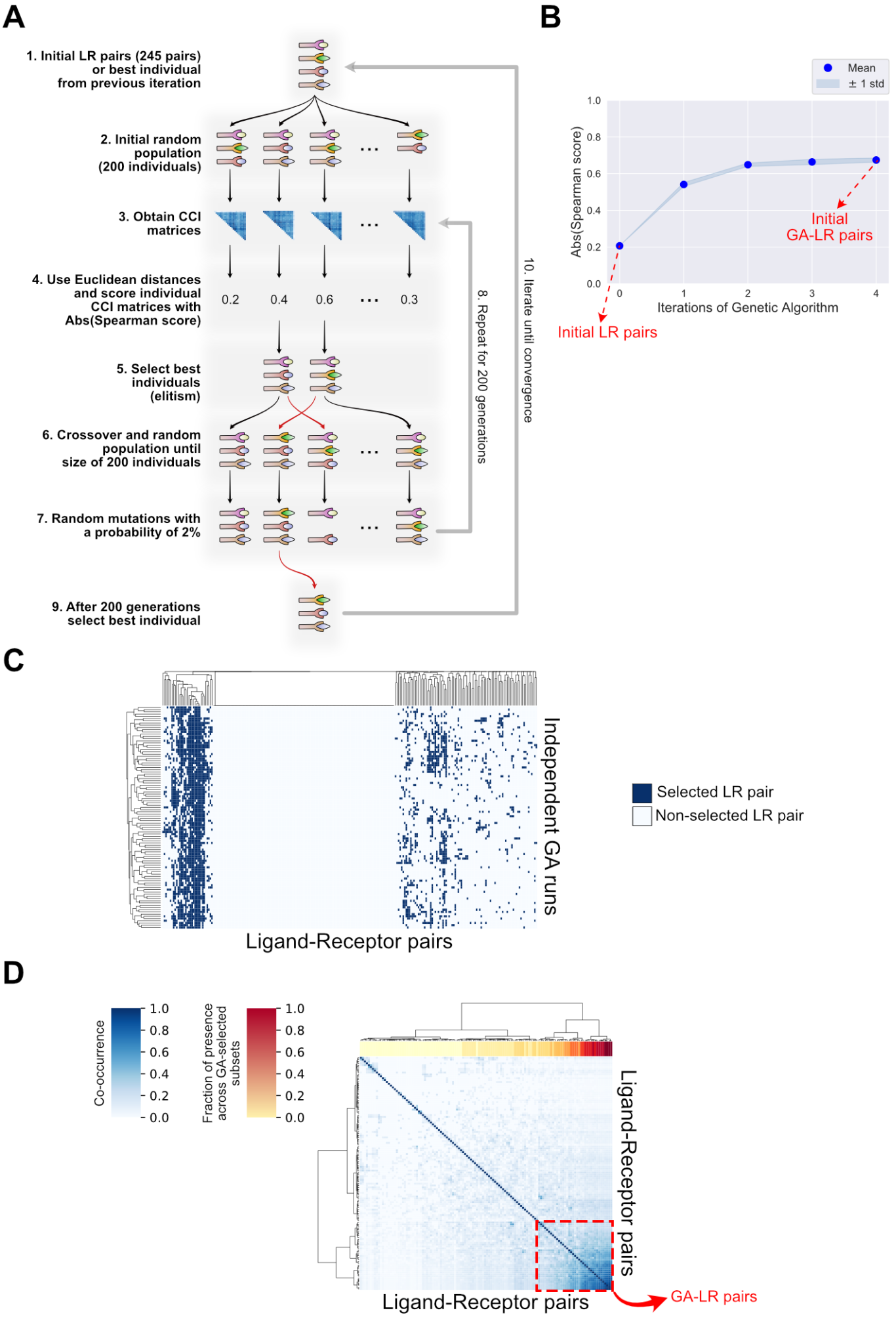


**N2 Fig. Genetic algorithm-based selection of ligand-receptor pairs leading to high CCI score-distance correlation.**

(A) Workflow for running the genetic algorithm (GA) and selecting a subset of ligand-receptor pairs leading to an optimal correlation between CCI scores and intercellular distances. Each run consists of ten steps described in the schematic representation. This framework was run 100 times independently, leading to 100 different subsets. (B) Absolute value of the correlation score obtained at each iteration of the GA. 100 independent runs are plotted, showing the mean and standard deviation across them. (C) Heatmap indicating when each of the 245 initial LR pairs was selected by one of the 100 GA runs. (D) Heatmap of the co-occurrence of two LR pairs. Only LR pairs that were selected in at least one GA run are shown here. The co-occurrence is defined as the number of runs that two pairs were selected together with respect to the total number when at least one pair was selected. The dashed red square represents the cluster that was finally selected as the consensus subset of LR pairs because of the high values of co-occurrence and high fraction of presence that its interactions had across the 100 GA runs.

**
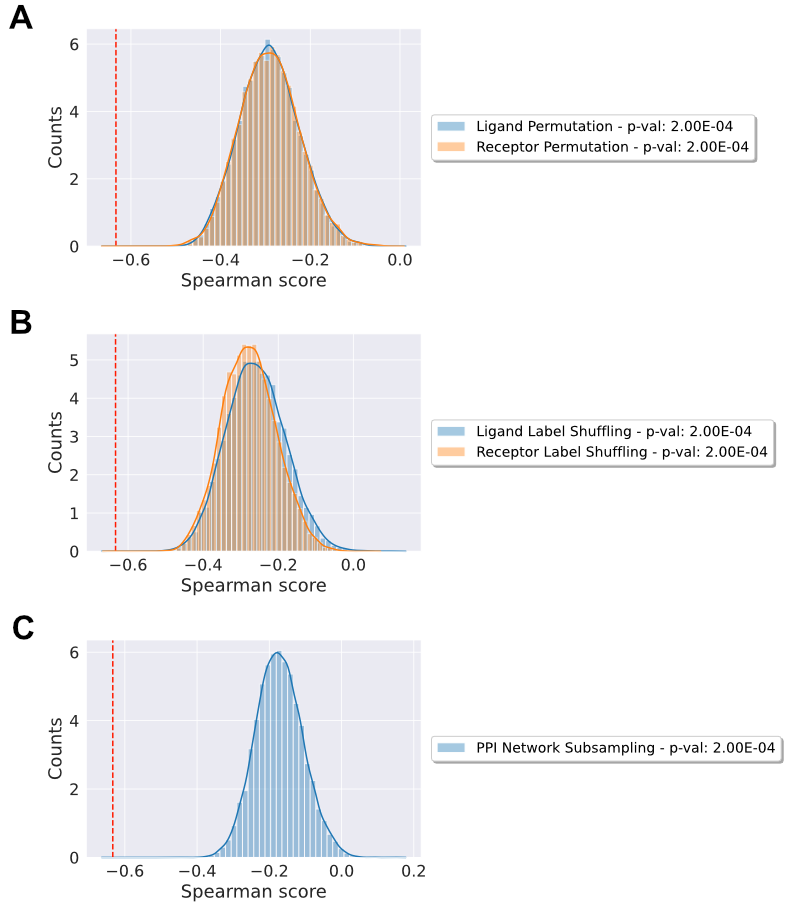
**

**N3 Fig. Permutation-based null distributions of the correlation obtained using the GA-LR pairs**.

(A) Null distributions resulting from column-wise random permutation of the interactors, either ligands or receptors, separately. (B) Null distributions resulting from random permutation of interactor labels, either of ligands or receptors, separately. (C) Null distribution resulting from random subsampling of the initial LR pairs (245 interactions) to generate subsets equivalent to the GA-LR pairs (37 interactions). Each analysis in (A-C) was run independently 10,000 times and for each run a correlation coefficient was computed, generating a null distribution in each case. In (A-C) the dashed red lines represent the correlation score obtained from the consensus GA-LR pairs.

**
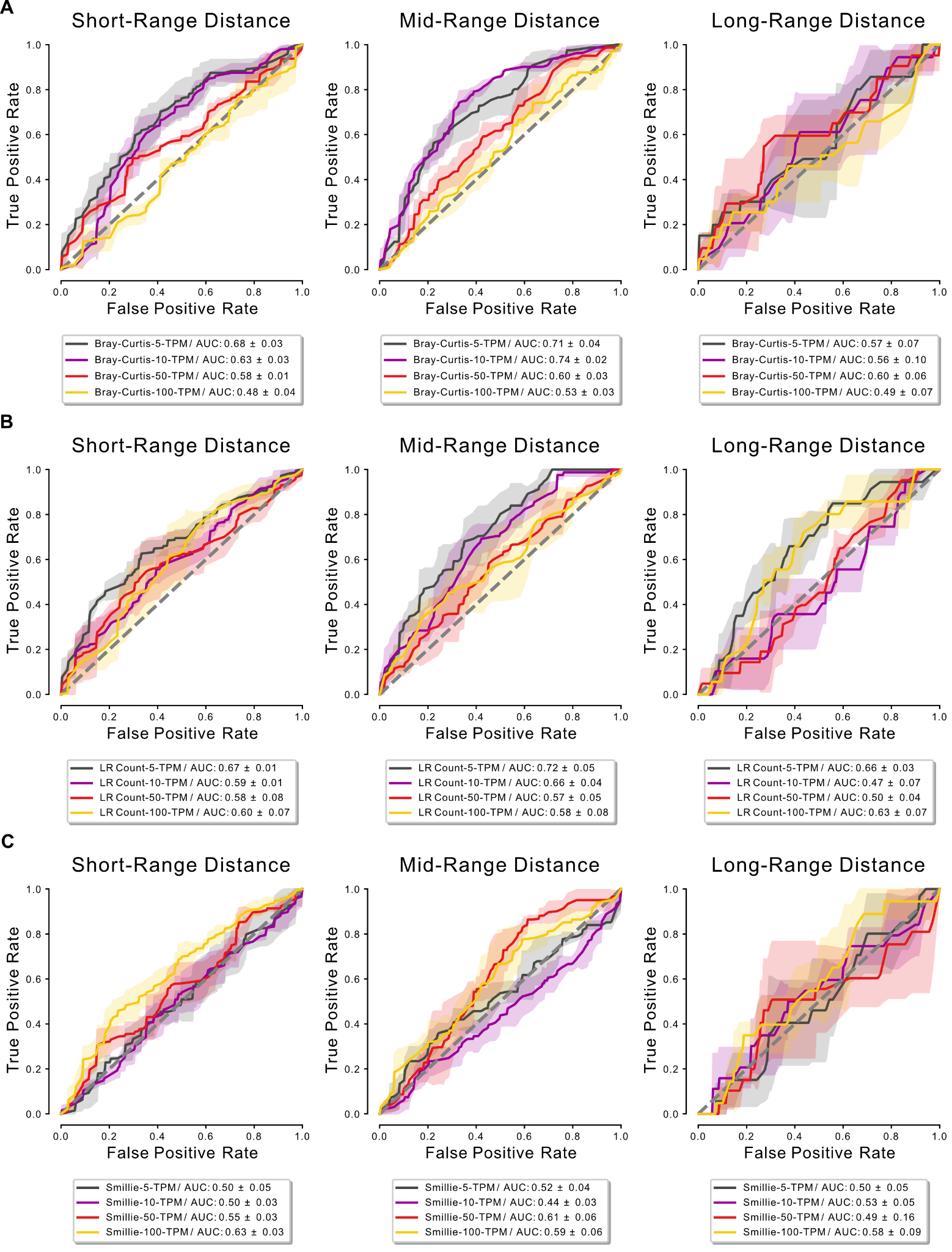
**

**N4 Fig. Benchmarking of CCI scores with different thresholds to assign presence of ligands and receptors**.

Receiver operating characteristic (ROC) curves of random forest models for classifying cell-cell pairs from their computed CCI scores. Here, we evaluated three different CCI scores computed from the binary presence of ligands and receptors, encompassing our Bray-Curtis score (A), the number of active LR pairs or LR Count score (B) and the Smillie score (C). Each of the CCI scores employed were benchmarked using different values of expression thresholds to assign the presence of ligands and receptors (5, 10, 50, and 100 TPM), as indicated in the legends. The classifiers predict the intercellular distance range (short-, mid-, or long-range distance, as defined in the N1 Fig). The performance is detailed through separate ROC curves for distinguishing each of the distance ranges from the rest. For each classifier, the mean (solid line) ± standard deviation (transparent area) of the ROCs were computed with 3-fold stratified cross validations. The area under the curve (AUC) for the ROC curves is shown in the legend below, detailing the mean ± standard deviation from the cross-validations.

# **Supplementary references**

1. [Ramilowski JA, Goldberg T, Harshbarger J, Kloppmann E, Lizio M, Satagopam VP, et al. A draft network of ligand-receptor-mediated multicellular signalling in human. Nat Commun. 2015;6: 7866.](http://paperpile.com/b/vrFqdk/e7mra)

2. [Cao J, Packer JS, Ramani V, Cusanovich DA, Huynh C, Daza R, et al. Comprehensive single-cell transcriptional profiling of a multicellular organism. Science. 2017;357: 661–667.](http://paperpile.com/b/vrFqdk/myXwx)
