# Supplementary figures and images for: Inferring a spatial code of cell-cell interactions across a whole animal body
Source: PLoS Comput Biol. 2022 Nov 17;18(11):e1010715. doi: 10.1371/journal.pcbi.1010715 (PMC9714814; doi:10.1371/journal.pcbi.1010715)

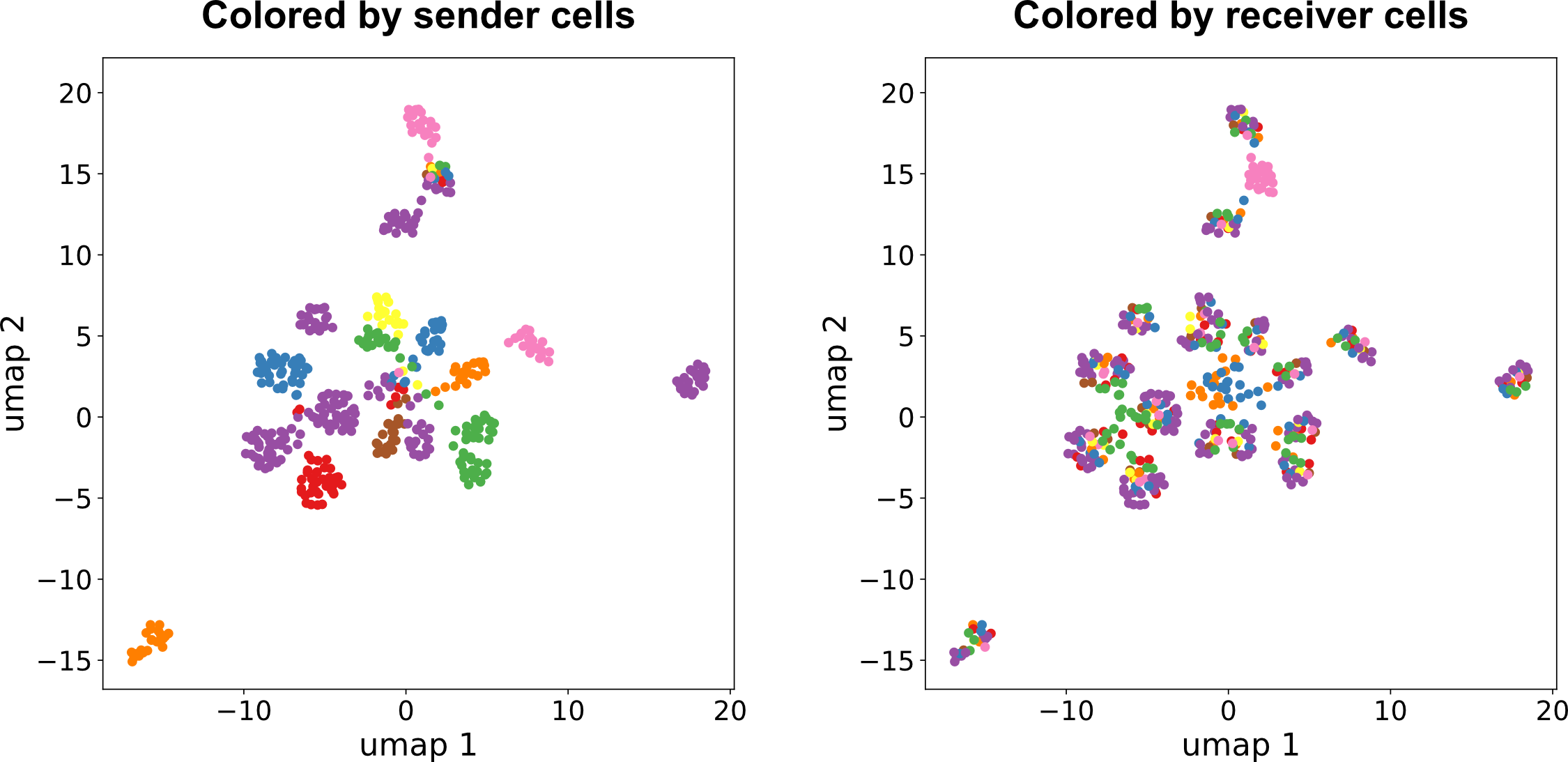

Supplement: S1 Fig — Visualization of the UMAP loadings computed for each pair of interacting cells. Dots represent pairs of interacting cells and they were projected based on their Rand distances (1-Rand index). In contrast to the Jaccard index that only accounts for true positives in the numerator, here the Rand index accounts for the true positives and negatives. It measures the number of agreements between two sets with respect to both the number of agreements and disagreements between these sets. Thus, the Rand index in this case was computed as the number of active and inactive LR pairs present in both cell types simultaneously, and divided by the total number of LR pairs in the database used (245 in this case, S1 Table). (TIFF) [file pcbi.1010715.s008.tiff]

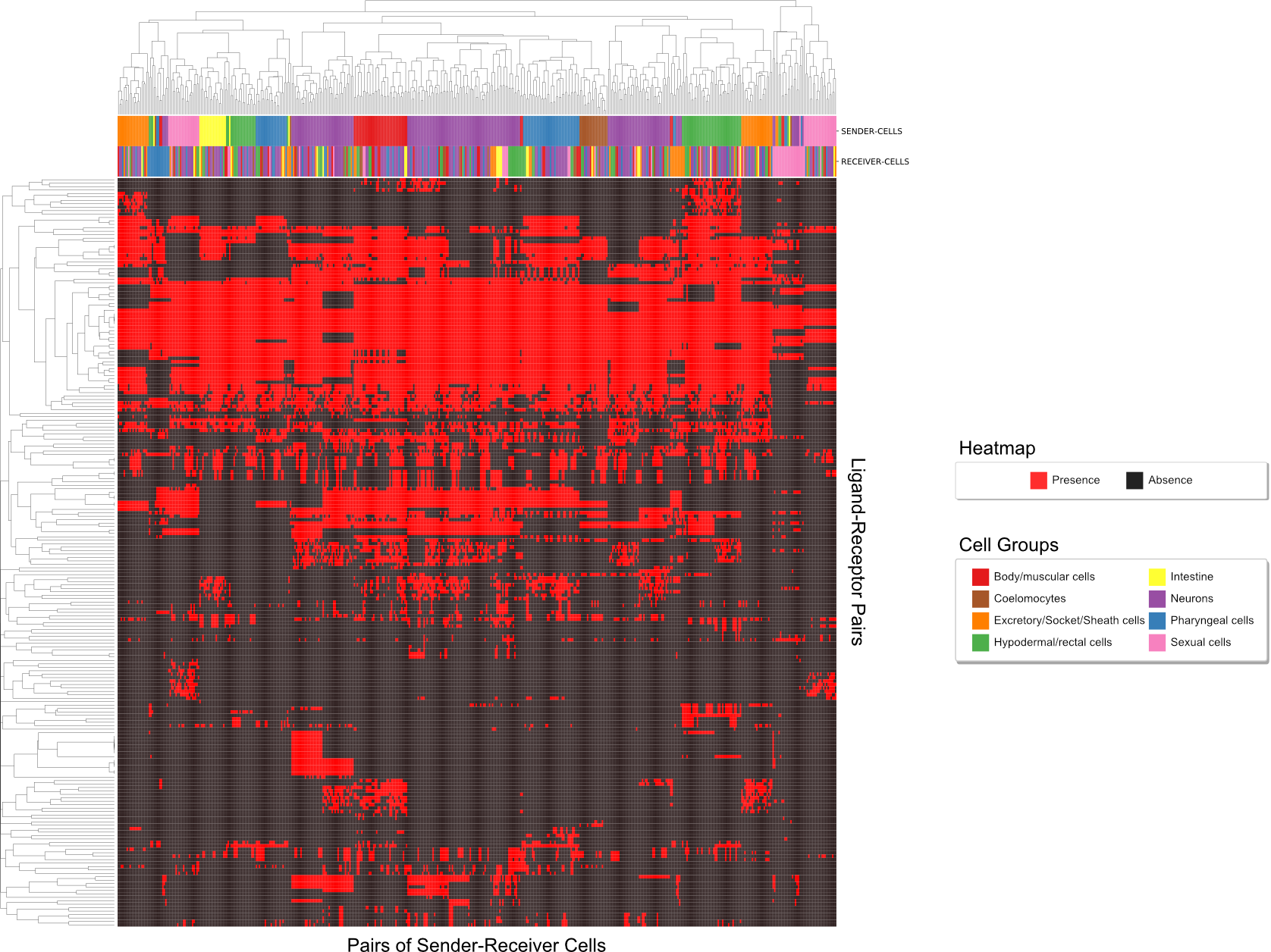

Supplement: S2 Fig — Heatmap of presence or absence of ligand-receptor pairs (y-axis) across all combinations of sender-receiver cell types in C. elegans (x-axis). An agglomerative hierarchical clustering was performed on the Jaccard similarity for the ligand-receptor pairs (dendrogram for rows) and the pairs of cells (dendrogram for columns columns). Additionally, sender-receiver pairs were colored either by the sender cell or the receiver cell, according to the groups in the legend. (TIFF) [file pcbi.1010715.s009.tiff]

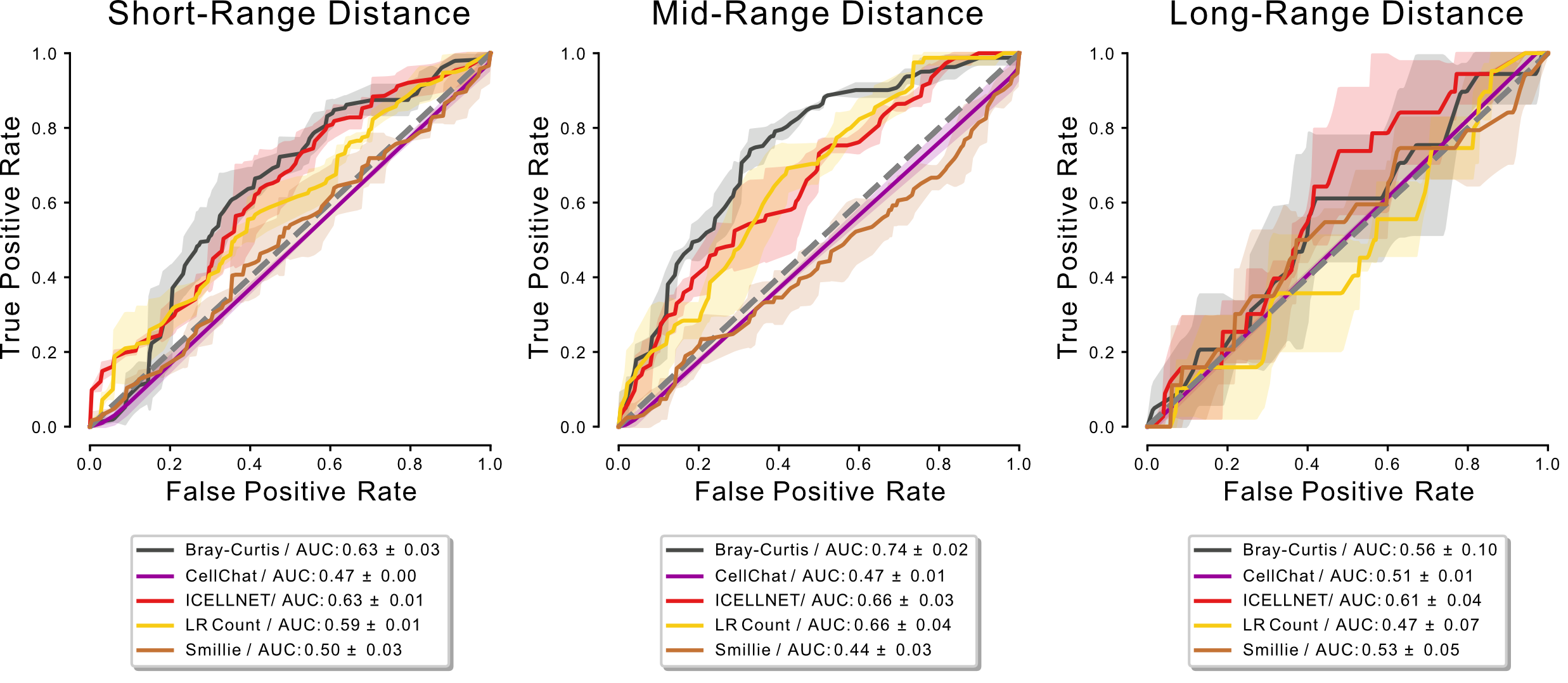

Supplement: S3 Fig — Receiver operating characteristic (ROC) curves of random forest models for classifying cell-cell pairs from each of the CCI scores computed with different methods, as indicated in the legends. The classifiers predict the intercellular distance range (short-, mid-, or long-range distance, as defined in the N1C Fig in S1 Text). The performance is detailed through separate ROC curves for distinguishing each of the distance ranges from the rest using each of the CCI scores. For each classifier, the mean (solid line) ± standard deviation (transparent area) of the ROCs were computed with 3-fold stratified cross validations. The area under the curve (AUC) for the ROC curves is shown in the legend below, detailing the mean ± standard deviation from the cross-validations. (TIFF) [file pcbi.1010715.s010.tiff]

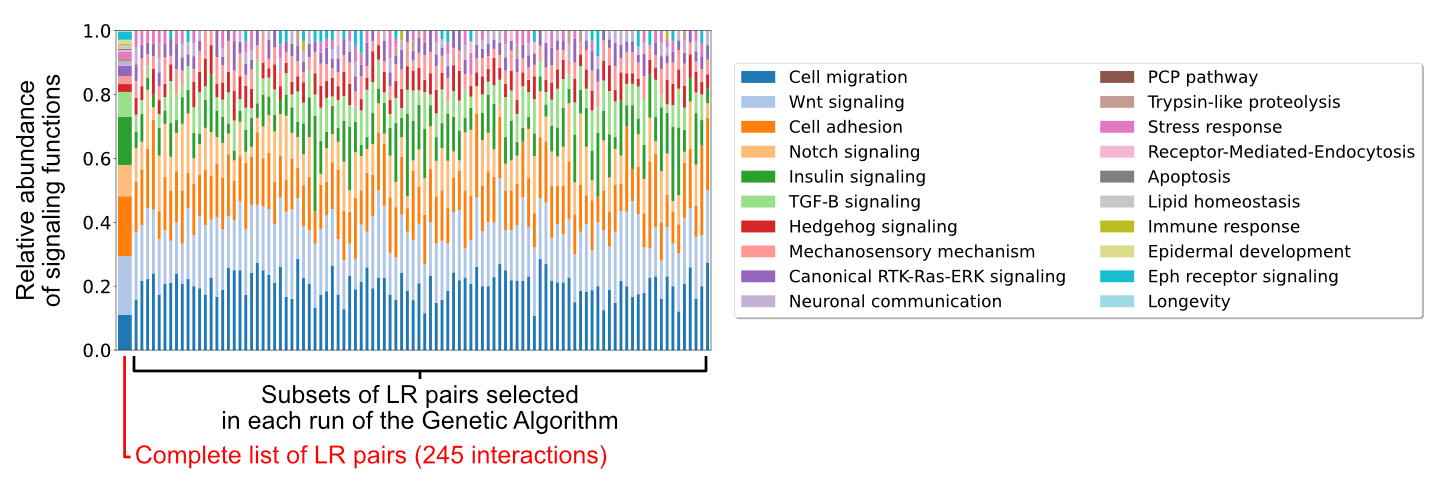

Supplement: S4 Fig — Composition plot given the signaling functions that LR pairs are associated with. Relative abundances are shown for the complete list of LR pairs (containing 245 interactions) and the subsets obtained in each of the 100 runs of the genetic algorithm (GA). Here, relative abundance is the number of LR pairs involved in a given pathway with respect to the total number of LR pairs in the list. Signaling functions are colored according to the legend. (TIFF) [file pcbi.1010715.s011.tiff]

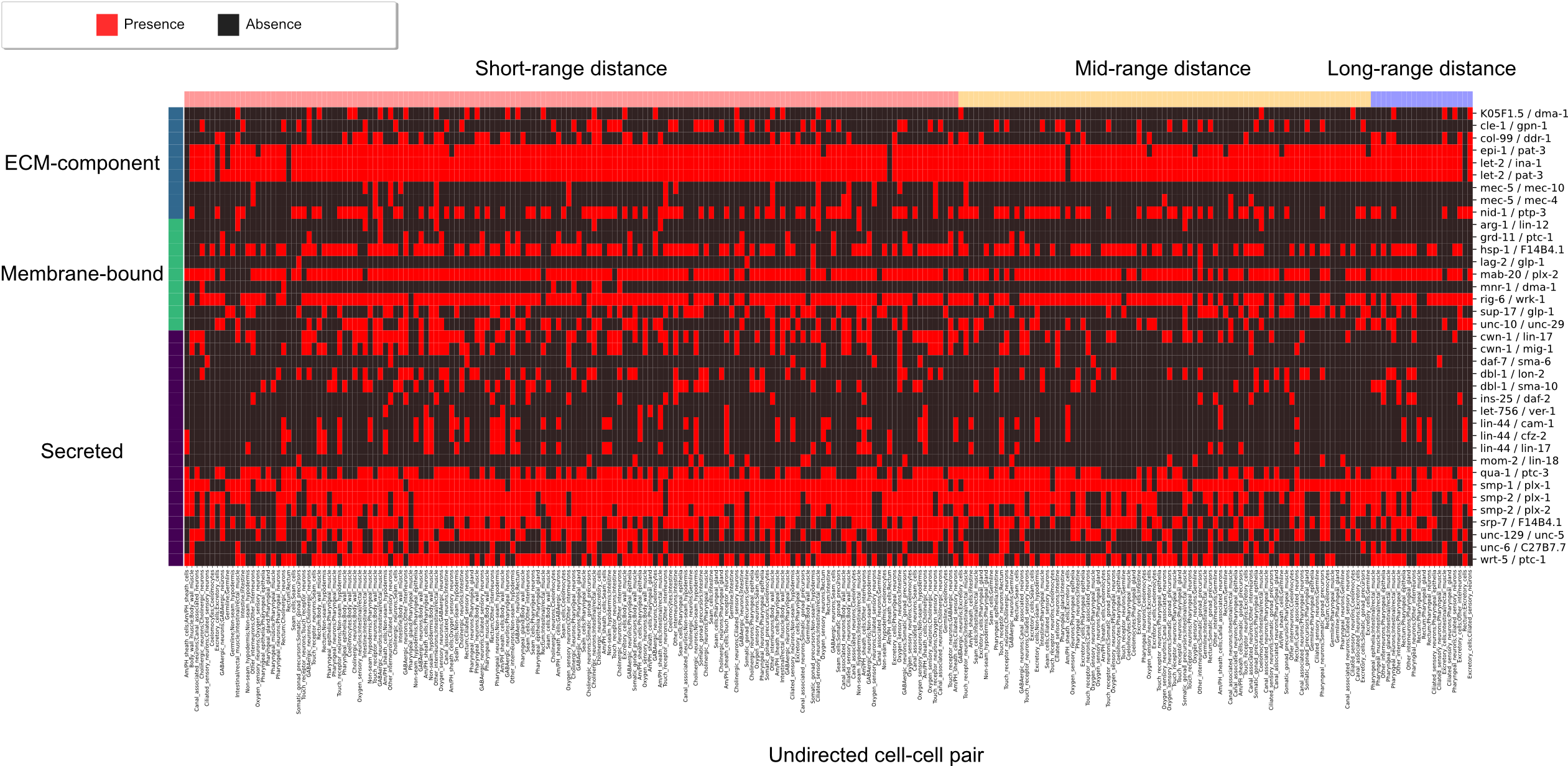

Supplement: S5 Fig — Heatmap of presence or absence of GA-LR pairs (y-axis) across all undirected cell-cell pairs in C. elegans (x-axis). Cell-cell pairs are sorted by their intercellular distances in an increasing manner, and are colored by the distance range as indicated above the colors (short-, mid-, and long-range distances, as defined in N1C Fig in S1 Text). Ligand-receptor interactions correspond to those in the list of GA-LR pairs, and each LR pair is considered present in an undirected cell-cell pair if it is used in either of the directed interactions between both cells. LR pairs are sorted and colored by the type of location where the ligand acts, as indicated to the left of the color (ECM-component, membrane-bound, or secreted). (TIFF) [file pcbi.1010715.s012.tiff]

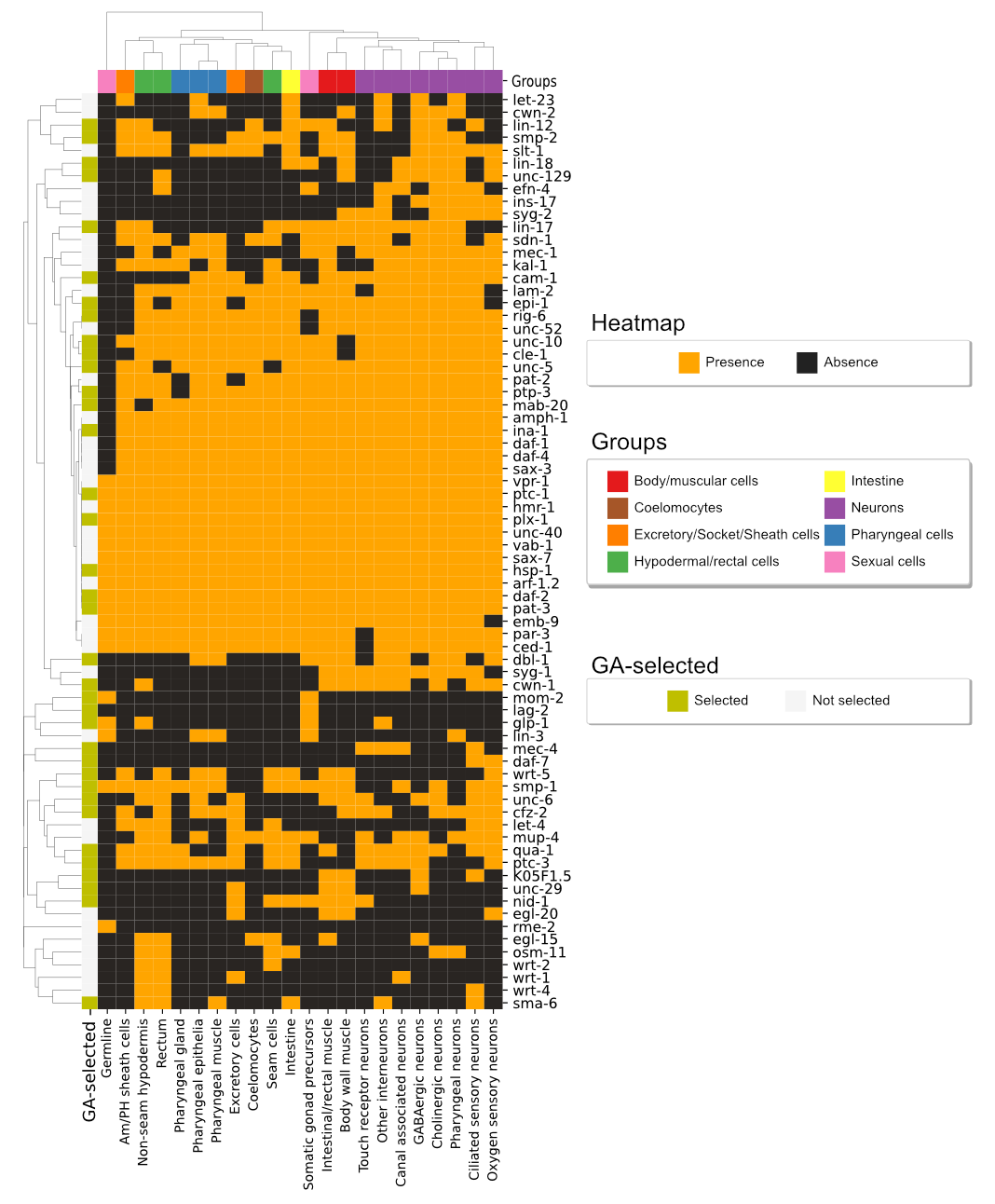

Supplement: S6 Fig — The presence or absence of proteins encoded by genes associated with organ system phenotype (y-axis) is indicated for each cell type (x-axis) according to C. elegans phenotype ontology. The threshold for presence is a gene expression value greater than 10 TPM; otherwise is labeled as absence. Only genes that are present in our complete list of LR pairs are shown, and members also in the GA-LR list are denoted with ochre cells (y-axis). Color keys for groups of cell types and GA-selection are depicted to the right. Agglomerative hierarchical clustering was performed using a Jaccard similarity for both genes and cell types, independently. (TIFF) [file pcbi.1010715.s013.tiff]

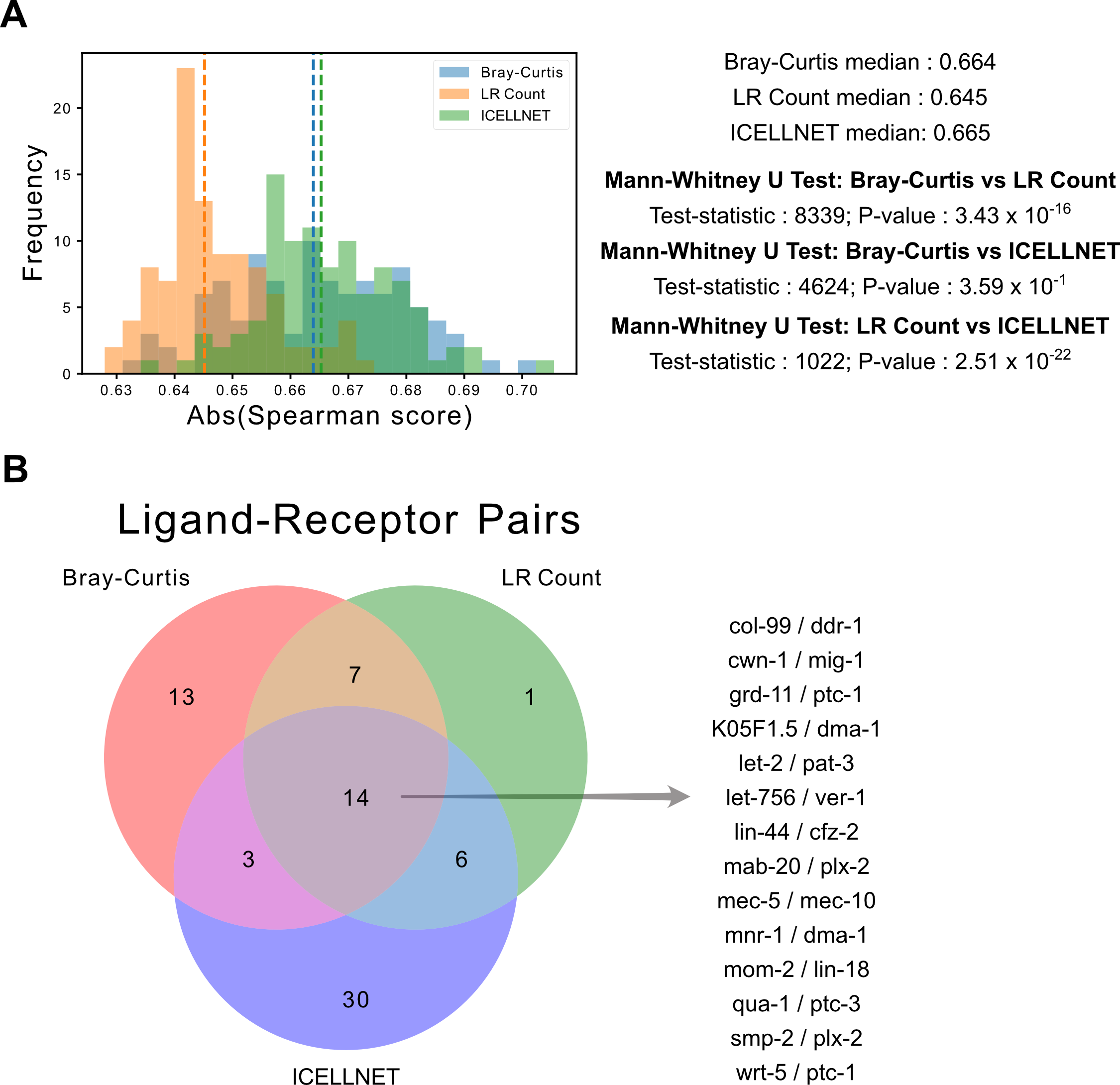

Supplement: S7 Fig — Comparison of running our computational framework by using the Bray-Curtis CCI, LR Count, or ICELLNET scores. (A) Histogram of the maximal Spearman correlation achieved in 100 separate runs of the genetic algorithm when using these CCI scores. The colors in the legend indicate which score each distribution corresponds to. Dashed lines represent the median values in each distribution. As indicated to the right of the histograms, a Mann-Whitney U test was performed to compare the distributions in a pairwise manner. (B) Venn diagrams of the LR pairs present in the consensus list of LR pairs for each of the CCI scores, obtained from the 100 separate runs of the genetic algorithm in each case. The list indicated by the arrow shows the LR pairs that are contained in all consensus GA-LR pairs (intersection between GA-LR pairs of Bray-Curtis, LR Count and ICELLNET scoring methods). (TIFF) [file pcbi.1010715.s014.tiff]

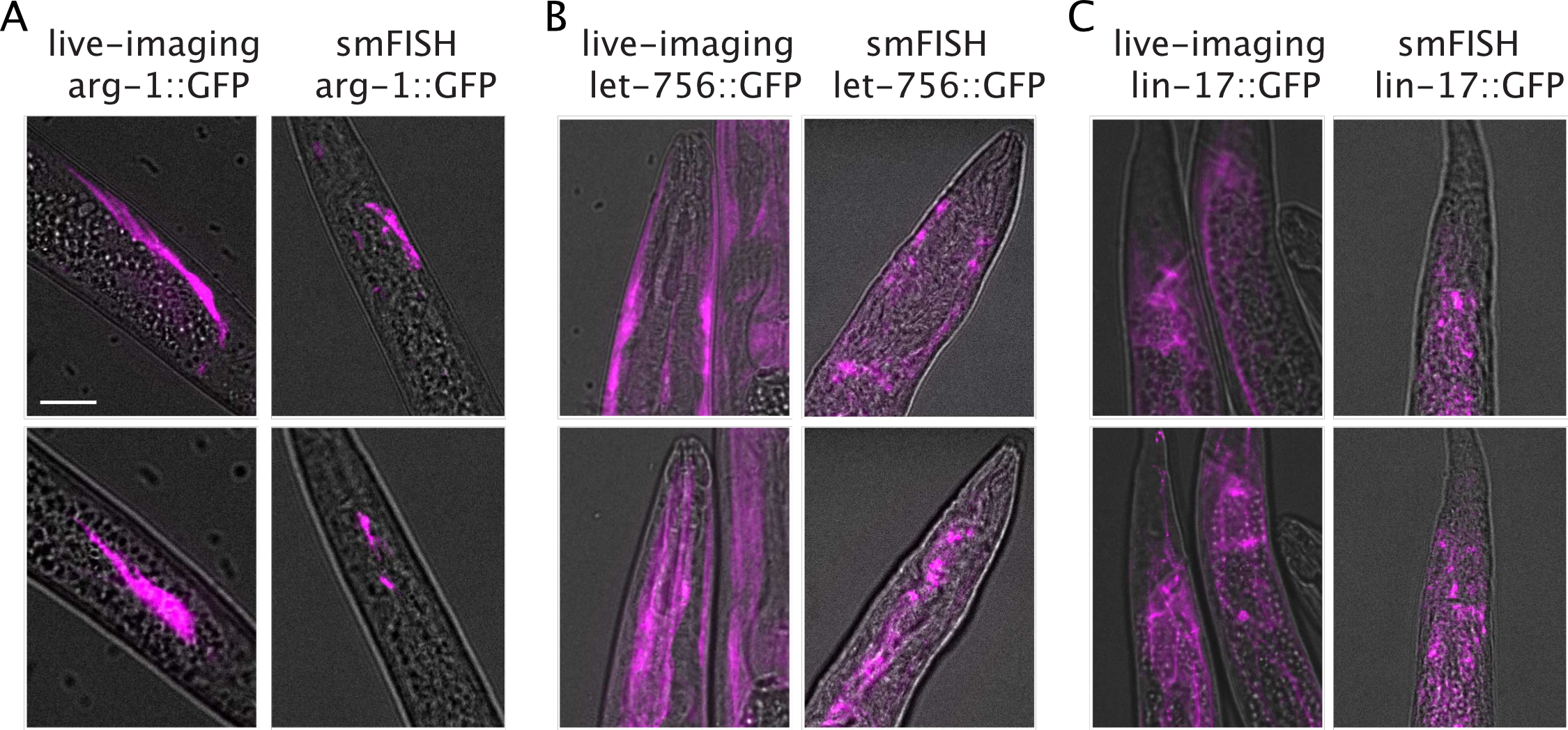

Supplement: S8 Fig — Expression patterns observed with smFISH overlap with those observed by live imaging of GFP, (A) arg-1 expression in the rectal muscle in both smFISH and live imaging, (B) let-756 expression in the non-seam hypodermal cells of the head in both smFISH and live imaging, (C) lin-17 expression in the tail seam cells in both smFISH and live imaging. In all cases we changed the colors of the original images into magenta to make the visualizations comparable. Scale bar = 10μm. (TIFF) [file pcbi.1010715.s015.tiff]

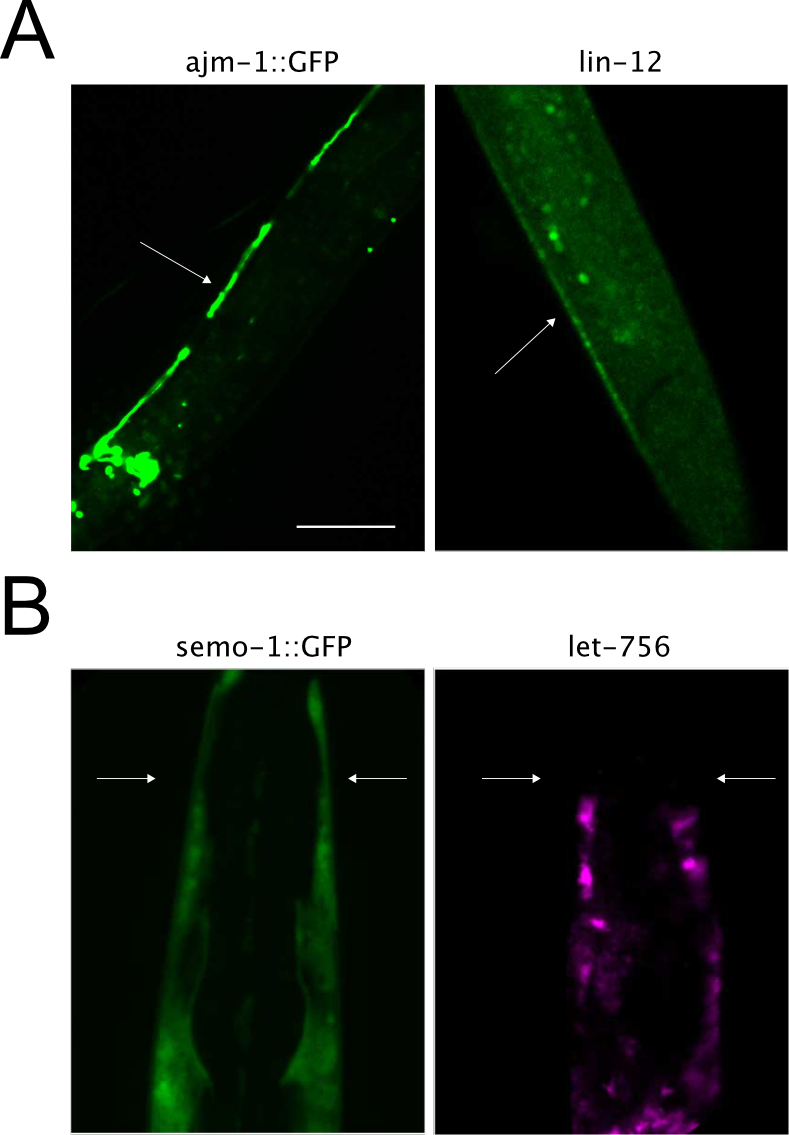

Supplement: S9 Fig — The expression patterns of lin-12 in the tail (A) and let-756 in the head (B) overlap with the expression patterns of ajm-1 in the tail and semo-1 in the head, confirming that the cells expressing lin-12 and let-756 in these regions correspond to non-seam hypodermal cells. Scale bar = 10μm. (TIFF) [file pcbi.1010715.s016.tiff]
