# Supplementary material for: Inferring a spatial code of cell-cell interactions across a whole animal body
Source: PLoS Comput Biol. 2022 Nov 17;18(11):e1010715. doi: 10.1371/journal.pcbi.1010715 (PMC9714814; doi:10.1371/journal.pcbi.1010715)
Supplement: Attachment — Submitted filename: Response-to-reviewers.docx [file pcbi.1010715.s020.docx]

# Reviewer #1:

Deciphering the cell-cell interaction mechanisms for spatial organization and cellular functions is important. Here the authors presented a computational framework, cell2cell, to infer the spatial code of cell-cell interactions from scRNA-seq data. The key component of cell2cell lies in a newly defined cell-cell interaction score based on the coexpression of ligand-receptor pairs. An interesting part was the identification of a subset of ligand-receptor pairs that were strongly anti-correlated with distance via a genetic algorithm. The authors demonstrated the performance of cell2cell by leveraging a 3D atlas of C.elegans’ cells and experimentally validating certain predictions. Overall, this study is very interesting and well suited for publication in PLoS Computational Biology. Below are a few comments that need to be addressed before publication.

We thank the reviewer for the positive comments. We think that the reviewer's input definitely helped to improve our manuscript. Below we provided detailed responses to each of their comments.

**1. The interaction score defined in this study was binary, which was different from the traditional methods that used the expression level of ligands and receptors. Can the authors provide a comparison analysis along with concrete examples to show the advantage of the binary communication score? Since many existing methods such as CellChat were based on the expression levels of ligands and receptors, it is very helpful if the authors can discuss the advantage and disadvantage of these two different scoring strategies.**

This is a very important point. Hence, we have added discussion about using binary vs continuous communication scores to build up an overall CCI score (Lines 421-426). The main advantage of binary scores is their ease of interpretation at the expense of losing resolution, while the main advantage of continuous scores is their higher resolution to distinguish different levels of importance at the expense of introducing bias into highly expressed LR pairs and losing interpretability. Furthermore, we included two new CCI scores based on continuous communication scores (CellChat and ICELLNET, which rely on mass-action principles and expression product, respectively) to perform a comparison between different kinds of overall CCI scores (see Lines 176-195). Particularly, we contrasted the ability of the different scores to be used for classifying intercellular distance ranges from the overall CCI score for each pair of cell types (Fig. 2C). Permutation-based scores were the ones performing the worst for the application here, but we discussed that this may be due to the purpose of these scoring methods –to find interactions that are cell-type specific, helping to deal better with false positives (Lines 189-193). Further discussion is provided in our Discussion section (Lines 416-432).

**2. The spatial transcriptomics is growing and there are many spatial transcriptomics datasets now. Due to the special body size of C. elegans, is the proposed method applicable to spatial transcriptomics of other tissues. The spatial location information from spatial transcriptomics data may be better to assess the correlations between the defined scores and the spatial distance.**

We appreciate your comment regarding the use of our method with spatial transcriptomics data. Our method is indeed applicable to this kind of data, but there are some limitations with using our approach with spatial transcriptomics technologies. This includes, for example, the low number of detected genes depending on the spatial technology, limiting the number of LR interactions that could be studied; and the detection of tissue spots, limiting the study of single-cell or requiring a deconvolution of the spot composition (which may propagate error associated with the deconvolution). Nevertheless, we had explored the possibility of including a study case with these technologies, but we did not find an adequate dataset for *C. elegans* that contains both cell coordinates and the gene expression levels in a comprehensive manner. These datasets exist for tissues of other organisms, and could be the basis of an exciting follow up study. Indeed it should be a separate study since the results would require validations that are out of the scope of this work. We have, however, highlighted this potential application with a couple of sentences in the Discussion section (Lines 494-496).

**3. The interaction score was defined for each pair of cells. A computational cost issue may raise when the number of cells was very large. For example, it may be challenging to project the cell-cell interactions onto a UMAP space like Fig. 2B.**

Thanks for pointing out this concern. Higher number of cells could quickly increase the number of cell pairs and therefore the demand of computational resources, which could be a problem when dealing with single cells. However, as occurs in most of the tools for inferring cell-cell communication, we deal with cell types aggregated from the corresponding single cells, which substantially reduces the number of cell-cell pairs into a number that is easily handled by our tool and analyses such as UMAP. In cases with a high number of cell types that may make the analysis computationally expensive, one could subsample the cell pairs, very similar to the way that tools designed with single-cell resolution handle this problem (e.g., NICHES <https://doi.org/10.1101/2022.01.23.477401>).

# Reviewer #2:

In their manuscript “Inferring a spatial code of cell-cell interactions (CCIs) across a whole animal body”, the authors present a novel method for quantifying the degree to which cell types interact, inferred from their transcriptomes, then make the argument that the ligand-receptor (L-R) pairs that contribute to the relationship between intercellular interactions and their physical distance are involved in defining the tissue morphogenesis that drives that spatial arrangement. The major contributions of this work are the novel CCI scoring method, and that previously unknown roles in tissue organization can be inferred for L-R pairs that contribute to the relationship between interaction strength and physical proximity in cell type pairs.

The first contribution is of relevance to the field of single-cell transcriptomics, where it has become popular to infer CCIs from gene expression data. While this paper makes no novel contribution to the subgenre of that field interested in predicting the individual L-R pairs involved in these interactions, the modified Bray-Curtis dissimilarity this work proposes to quantify the relative “strength” of interactions between pairs of cell types is novel - albeit similar in goal (specificity of interaction) to Smillie et al. (<https://doi.org/10.1016/j.cell.2019.06.029>), where strength of CCIs were quantified as the probability of seeing as many L-R pairs between cell types by chance as calculated by a Monte Carlo simulation. No metric for CCI strength has been adequately evaluated experimentally in the current literature, so it is difficult to compare the author’s modified Bray-Curtis score to existing measures. However, the authors propose a novel evaluation metric by hypothesizing that interacting cells are in physical proximity, and thus CCI scores may be evaluated based on their relationship with physical distance between cell types. Taking advantage of the stereotypic arrangement of C. elegans cells and its recently published single-cell transcriptomic atlas, the authors are able to show that the modified Bray-Curtis score does reasonably well by this measure, showing a weak relationship between interaction strength and distance. I applaud the authors for this creative application of existing knowledge to validate their new metric.

We thank the reviewer for the kind comments and the useful feedback to help strengthen our analyses and manuscript. Below we provided detailed responses to each of their comments.

**1.** **I would suggest that given their argument for the importance of a CCI score that captures specificity, as well as the similarity of their score to Smillie’s, the authors should consider using their metric to evaluate their score in the context of both Smillie’s statistical measure, and the commonly applied sum of L-R pairs as a CCI score. I’d expect their score to perform better than the sum of L-R pairs, and similar to Smillie’s. The advantage of the modified Bray-Curtis over Smillie’s statistical test is its simplicity, so as long as performance is similar, it would be fair to conclude that the Bray-Curtis score has an advantage.**

We thank the reviewer for this suggestion, and we think this suggestion adds new perspectives to the analysis of intercellular spatial properties. We implemented the suggested score, and compared it with our Bray Curtis score, the LR count score, and CCI scores based on CellChat and ICELLNET (Fig. 2C and text in Lines 176-195). However, the Smillie score is based on permutations, similar to CellChat, which makes this kind of score intended to find cell-type specific properties of the interactions, helping to provide novel insights about cell-cell communication (we now mention this in our manuscript as well, Lines 189-193). When considering spatial properties of communication, it’s also important to consider all mechanisms that could contribute to these properties, even cases that are preserved or in common across cell types. This information is lost with the CellChat- and Smillie-based CCI scores, which could explain why both scores based on permutations resulted in the lowest performance on separating different intercellular distance ranges of interaction.

**2. My only concern is that the metric relies on accurately inferring L-R pairs which interact between cell types, something that is not a solved problem (I appreciate that the authors did note that these methods only have the ability to predict what L-R pairs could be used, not necessarily which are actually being used). Given that L-R interaction is inferred by expression over an arbitrary abundance threshold in this work, it would be interesting to see how changes in that threshold affect the correlation between CCI strength and physical distance. For example, if the expectation is that ligands expressed below threshold aren’t contributing to signaling, as the threshold is lowered the correlation may become weaker. Ultimately, the authors have proposed a novel cell-cell interaction prediction method cell2cell, and a creative solution to provide supporting evidence for its efficacy, and I’d like to see that used to demonstrate that the current parameterization of cell2cell is optimal, even if comparing cell2cell to other existing methods for inferring ligand-receptor interactions is beyond the scope of the paper.**

We appreciate the reviewer for bringing up this concern, since it helps us to make our work as comprehensive as possible in terms of computing the score. We compared different scenarios of threshold values and extended the discussion in the section *Threshold values for the binary-based CCI scores* of the Supplementary Notes, and provided a new figure contrasting them (Fig. N4 of the Supplementary Notes). However, it’s important to consider that optimal parameterization could lead to an overfit when looking for correlations between the CCI scores and the phenotypes. To avoid this, we chose the threshold value of 10 TPM as selected in Ramilowski et al (<https://doi.org/10.1038/ncomms8866>), which is a value that optimizes true negatives and false positives in terms of the real presence of a protein (discussed in the Supplementary Notes). Nevertheless, we developed cell2cell to be flexible on how scores are computed and leave the freedom to the user to input the parameters that they consider the best for their own purposes, instead of using optimal parameterization of our tool.

**3. The second contribution of this work is its method for hypothesis-generation of novel morphological roles for L-R pairs. With the rise of spatial scRNAseq, this could become a popular method for identifying candidate signals involved in tissue morphogenesis. My only question is whether the use of a complicated genetic algorithm was necessary in this case, as ultimately the spatial code was (at least in part) defined by relative enrichment of L-R pairs in each body section. Given that the genetic algorithm is the most computationally expensive part of the analysis, it would be interesting to see how skipping it (passing all L-R pairs to the spatial enrichment analysis) or using a less powerful but presumably quicker method to select L-R pairs driving the CCI-spatial correlation affects the efficiency of this method for generating accurate hypotheses regarding L-R pairs involved in morphogenesis. That being said, I appreciate that validating these hypotheses was not trivial, so consider this a suggestion for improving the general utility of the method rather than a requirement for publication.**

Thanks for bringing this point. It is true that the genetic algorithm (GA) is more complicated and computationally expensive than other methods. Indeed, when delineating the analyses to perform in this work, we were thinking of using a different approach. One simple option was a linear regression with the communication scores as variables and intercellular distances as outputs to obtain coefficients for each LR pair and their potential importance for the spatial proximity of cells. However, in this case we ran into the problem of the directionality of cell-cell pairs. We would have, for example, a LR-X in two data points: the directed interaction between A->B and the interaction B->A, while the intercellular distance is still the same for both data points. One solution could have been putting all LR pairs of each data point involving the same cells together in one data point, but the column order of the LR-X(A->B) and LR-X(B->A) in the input matrix would be important when accounting for other cell-cell pairs since order alternation across data points may affect the coefficients of these variables when performing the regression. For this reason we discarded this option. The enrichment analysis could have also been an option, but limited to categories of intercellular distances or body regions instead of continuous values of distances. Furthermore, when considering the spatial code as a subset of the whole list of LR pairs explaining intercellular distance, we do not know beforehand the size of this subset. The enrichment analysis could lead to LR that are enriched/depleted but not necessarily into a subset that their simultaneous activations/inactivations explain the intercellular distance. For this reason, a search among different subsets of varying sizes is necessary. In that case we end up with a universe of $\sum_{i=1}^{d} \left( d choose i \right)$or (2^d)-1 potential subsets to explore; where d is the number of LR pairs in the complete list, which in this study corresponds to 245 LR pairs, and therefore ~10^73 subsets to explore. For that reason, the GA was a plausible choice to quickly obtain an optimal subset to better represent the intercellular distances through an overall CCI score. Nevertheless, other options could be explored to help speed-up the analyses when larger datasets are analyzed. We added a couple of sentences in the discussion mentioning this (Lines 506-509).

**4. The GA-LR result contains a consensus, but it would also be interesting to know how much redundancy there is in the set. Is there a lot of redundancy in general in the network? Could this be linked to robustness?**

We agree with the reviewer that redundancy in the network is an important concept to explore since cells could use alternative pathways for a given function. However, from the options we evaluated we are not able to offer a clear answer to this. We analyzed redundancy in the functions of LR pairs and how cells could use them; redundancy in terms of different ligands using the same receptor, and vice versa; redundancy in terms of LR pairs that are excluded from others (only one of the LR pairs with a similar function is present in a given run of the GA; e.g. using information in Figs. N2C-D); and redundancy in the gene expression of the ligand and receptor genes (e.g. using information in Figs. 4B, S5, and S6). It would also be interesting defining a metric to link it to robustness, but unfortunately we were not able to identify a clear behavior to come up with measurement. Somehow Fig. S7 could represent robustness of the GA in finding the correlation with intercellular proximity, and in selecting LR pairs regardless of the CCI score employed. Anyway, future work in exploring this kind of properties in cell-cell interactions would be definitely important.

**5. It is not clear what exactly the spatial code is and how strong it is. For example, is the spatial code just a correlation? And what use does this correlation have? Can we use it to improve LR interaction prediction? Or is it a skeleton physical network that matches animal anatomy? If the latter is the case, then you may be able to reconstruct aspects of the organism body plan based on the spatial code directly from scRNA-seq data. Is this possible? If we are to interpret the code directly as physical/biochemical, then additional questions may be raised that would be useful to explore to support the concept. For example, are cell adhesion (e.g. integrin, cadherin) relationships more likely to be expressed in nearby space compared to paracrine interactions?**

This is a crucial question since a good definition is important for the readers to better understand the purpose of our work. Therefore, we have improved the definition of the spatial code in both the abstract and the introduction (Lines 56-60). Here we looked for a subset of LR pairs better explaining intercellular distance (resulting in the GA-LR pairs), a relationship that was represented through the correlation between the CCI scores and the intercellular distances. Thus, this subset is intended to make the CCI score to better represent intercellular proximity, and therefore this CCI score to be used as a skeleton physical network matching animal anatomy. As the reviewer suggested if that was the case, we added new analyses to interpret the spatial code, besides the analyses that previously did (e.g., Figs. 3 and 5). For example, we evaluated the enrichment/depletion of ECM-component, membrane-bound, and secreted LR pairs in the different distance ranges between cell types (Lines 311-329 and Fig. S5). Additionally, we ran the GA on other CCI scores beyond our Bray-Curtis score to obtain their corresponding “spatial codes”, and found a good overlap between all consensus lists (Fig. S7), regardless of the different purposes of each CCI score method (discussed in Lines 427-432).

**6. The manual curation is suspicious and should be double-checked. e.g. F14B4.1 does not seem to be involved in CCI. It is labeled as a Wnt-receptor in table S1, but I can't find evidence of this in wormbase. There are a number of interactions with hsp-1 - are these all real CCIs? Is hsp-1 accessible to the exterior of the cell?**

We thank and value that the reviewer double checked some of the interactions in our curated database of ligand-receptor pairs. In our DB we considered LR pairs that are orthologs to LR pairs in human DBs, and kept pairs that are consistent with suggested roles of their interacting partners. A few cases included in our DB lack robust information since some of them are putative in the role they play. The description we provided for each LR pair is based on the WormBase version WS277 from 2020, so it may change in posterior versions. To avoid this kind of concern, we adapted our DB and specified the version of WormBase that we used to provide the description of the interactions. In addition, we went over each LR pair and annotated them either as 1) putative based on data (present in PPI DBs and orthology information), 2) putative based on literature (same as category 1 plus the fact that its putative role has been described in literature), and 3) literature supported (its role has been demonstrated in literature), so researchers can decide what level of confidence of the LR interactions to use (see new columns “Pair_confidence” and “Confidence_evidence” in the table S1). From this annotation, we have that from the interactions: ~9% is putative based on data, ~17% is putative based on literature and ~74% is literature supported. Furthermore, we added another column (“Location_type”) to indicate whether the LR pair is involved in membrane-bound, secreted, or ECM-component interactions.

The case of F14B4.1 was annotated to have a role in the WNT pathway in 2020, but that information was changed in 2021 to another putative role that is not associated with cell-cell interaction. However, literature suggests it has a role as receptor (<https://doi.org/10.1523/JNEUROSCI.3237-15.2015>) so we kept our initial annotation for this case and added the annotation of “putative based on literature”. In the case of hsp-1, its interactions are based on the role in cell-cell communication of its human ortholog HSP-70 (<https://doi.org/10.1017/s0031182012002168>, <https://doi.org/10.1016/0378-1119(88)90339-3>), which can be secreted (<https://doi.org/10.2174/1389203715666140331113057>), potentially participating in intercellular interactions (annotated as “putative based on literature”). Also, hsp-1 has been annotated to localize in the plasma membrane (<https://www.uniprot.org/uniprotkb/P09446>), suggesting a role in membrane-bound cell-cell interactions.

## Minor notes:

**The use of “spatial code” in the abstract / author summary is a little hard to follow as the concept hasn’t been adequately defined for the reader yet. By the end of the intro, the goal of the paper is clear, but in the abstract it might be necessary to spend a few more words than “spatial code” elaborating on the paper’s objective.**

We have now introduced what the spatial code is in the abstract.

**Fig 1: the visual attributes chosen are not consistently used. For example, the receptors have different colors (slightly, and difficult to differentiate) and the ligands don’t seem to. The shapes are different and that should be enough - it would be useful to simplify the shapes to make their complementarity easier to see. Another example is the purple to green gradient - it seems to be used for two things: one to distinguish the cells (that’s ok), and the other to highlight the difference between headings and data in the middle of the figure. Purple is further used for a few other shading areas that seem unrelated to the cell identity.**

We thank the reviewer for this suggestion. We have improved the figure accordingly.

**Fig2b: Jaccard score seems biased toward sender cells. While this does reflect previous findings and could be due to biology, Jaccard index can be biased in the case of class imbalance (because the numerator only considers true positives, ignoring true negatives) and the LR database contains twice the number of ligands as receptors. To defend against that critique, this analysis bears repeating with a different similarity metric better suited to imbalanced classes (perhaps adjusted Rand index).**

This point is relevant since there is indeed a class imbalance. We have now performed the comparison taking into account the true negatives as suggested, and generated a new UMAP based on the Rand metric (Fig. S1). However, we still observe the same behavior as using the Jaccard metric, suggesting that the behavior could be due to biology. We kept the original UMAP as the main figure since readers may be unfamiliar with the Rand index, but mentioned in the figure caption that the Rand index is more appropriate for this purpose and pointed them to the supplementary figure.

**Fig3a: Not very informative - the authors are trying to indicate changes in relative abundance of signaling functions per GA run output, but unfortunately it is difficult to follow. Perhaps 3a can be enlarged in the supplement for anyone skeptical about consistency between runs, and Fig3 can be a volcano plot of relative change (magnitude, x-axis) vs. significance (-log10 adj. p-value), which would be more effective at visually conveying the conclusion of Fig3b without getting bogged down in trying to represent each GA run.**

We have replaced Fig. 3 by boxplots showing the fold change level of the relative abundances for each LR function across all GA runs. We also added the old Fig. 3A as the new Fig. S4.

**Fig 4b: Perhaps matrix columns should match Fig4a for clarity?**

Thanks for this idea, we have changed the order.

**“Thus, we hypothesized these PPIs represent a spatial code, that when used in different combinations can encode different spatial proximities.”**

**The combination claim is not supported**

This has been removed.
